# A Near-Peer Educational Model for Online, Interactive Learning in Emergency Medicine

**DOI:** 10.5811/westjem.2020.12.49101

**Published:** 2020-12-21

**Authors:** Hailey B. Rosenthal, Neha Sikka, Adam C. Lieber, Charles Sanky, Christian Cayon, Daniel Newman, Denisse R. Marquez, Jacob Ziff, James R. Blum, Jennifer B. Dai, Phillip Groden, Sara Pasik, Trevor Pour

**Affiliations:** Icahn School of Medicine at Mount Sinai, Department of Emergency Medicine, New York, New York

## Abstract

**Introduction:**

The COVID-19 pandemic led to a large disruption in the clinical education of medical students, particularly in-person clinical activities. To address the resulting challenges faced by students interested in emergency medicine (EM), we proposed and held a peer-led, online learning course for rising fourth-year medical students.

**Methods:**

A total of 61 medical students participated in an eight-lecture EM course. Students were evaluated through pre- and post-course assessments designed to ascertain perceived comfort with learning objectives and overall course feedback. Pre- and post-lecture assignments were also used to increase student learning.

**Results:**

Mean confidence improved in every learning objective after the course. Favored participation methods were three-person call-outs, polling, and using the “chat” function. Resident participation was valued for “real-life” examples and clinical pearls.

**Conclusion:**

This interactive model for online EM education can be an effective format for dissemination when in-person education may not be available.

## BACKGROUND

On March 17, 2020, the American Association of Medical Colleges issued guidance that encouraged schools to implement a two-week suspension of clinical activities for medical students due to the COVID-19 pandemic.^1^ This guidance was extended, and in-person clinical education for medical students was halted nationally for months.^2^ This interruption impacted medical students in countless ways, ranging from educational to professional development.^3^,^4^ Time away from clinical rotations was particularly disruptive for students applying into emergency medicine (EM) due to delayed exposure to the field, the historical importance of Standardized Letters of Evaluation (SLOE), and the unique effect of the pandemic on the emergency department.^5^

Recognizing the impact of this disruption on students interested in EM, the American College of Emergency Physicians, Emergency Medicine Residents’ Association, and Council of Residency Directors in Emergency Medicine issued a joint guidance encouraging schools to “explore novel programs to expose medical students to virtual clinical experiences.”^6^ This direction has been adapted across medical education through innovative approaches to online learning formats — from small-group teaching and specialty-specific clinical skills to online clinical bootcamps.^7^–^9^ In EM, remote learning has connected medical students to the clinical environment, while learning activities for residents have largely shifted online.^10^,^11^ These transitions have brought their own trials and opportunities.^12^,^13^

Given the challenges faced by students and the sudden shift to online education, there was an immediate demand to provide reputable options for interested individuals. This need presented an opportunity to bolster the education of these students through a novel online format. Previous work has evaluated curriculum in EM for third-year medical students, and the field has a rich tradition of engaging with continued learning through free, open access education (FOAM).^14^,^15^ As an educational model, case-based learning has been shown to successfully balance inquiry and structure, allowing students to efficiently accomplish case objectives while maintaining freedom for creativity and demonstrating problem-solving skills.^16^,^17^ Based on a review of the existing literature on medical education in EM, a peer-led, case-based online learning opportunity has not yet been evaluated as a means of educating medical students in their clinical years. We sought to create such a curriculum in the hopes of supporting medical students’ own education during this exceptional time while simultaneously assisting their peers with a shared interest in EM. Additionally, we sought to create a model that could be disseminated to other institutions, with the goal to fill this gap in clinical medical education.

## OBJECTIVES

The objective of the course, titled “Case-Based Approach to Emergency Medicine,” was to provide an interactive, digital modality to learn the basics of EM with fellow students and EM residents. This course was developed in April 2020 by and for medical students at the Icahn School of Medicine at Mount Sinai in New York City while much of the faculty were burdened with the significant increase in clinical responsibilities due to the COVID-19 pandemic. A group of 12 rising fourth-year medical students (MS4) organized the course and content under the supervision of the faculty mentor for this project, who serves as the Director for Undergraduate Medical Education for the Department of Emergency Medicine at the Icahn School of Medicine at Mount Sinai. In addition to guiding and approving important topics to include in the curriculum, this faculty mentor was also responsible for reviewing content prior to its use.

To identify appropriate topics for each lecture, the MS3 and MS4 topics from the Society of Academic Emergency Medicine’s (SAEM) Clerkship Directors in Emergency Medicine curriculum were reviewed and selected with the approval of the faculty mentor.^18^ Eight topics were identified and ultimately presented in the following order: EM imaging; chest pain and electrocardiogram (ECG); stroke and lumbar puncture; abdominal pain; altered mental status and toxicology; shortness of breath and ventilators; shock, sepsis, and intravenous fluids; trauma and the focused assessment with sonography for trauma (FAST) exam. Each lecture was created and presented by a team consisting of one or two medical students and an EM resident at their institution. Teams were responsible for the following: identifying two to three learning objectives to guide each lecture’s content; determining and providing pre-learning assignments; creating and presenting the lecture; and developing a post-lecture “homework” assignment. Lectures featured a short didactic followed by case-based discussions related to the lecture objectives. All lecture content and objectives were reviewed by the faculty mentor.

Pre-lecture assignments primed students for each upcoming topic and included podcasts, publications, clinical vignettes, and online content reviews. The lectures consisted of case reviews and didactics led by each team’s medical students, supplemented by residents who supplied real-life scenarios and clinical pearls. Lecturers engaged the class by asking questions throughout the presentation in a variety of ways that included the following: individual cold-calling (from a randomized roster list); cold-calling in groups of three; asking for volunteer responses in the video conference platform’s “chat” feature; using an online group polling software; and finally using the video conference platform’s “hand raise” feature. At the end of each lecture, the students were assigned homework to reaffirm their grasp of the material.

## CURRICULAR DESIGN

### Student Cohort

Recruitment for the course occurred approximately one week prior to the first lecture via an email sent out to medical students highlighting the course objectives, requirements, and schedule along with an attached sign-up sheet. In response to an overwhelming volume of students who expressed interest, and to preserve a highly participatory learning environment, a cap of 50 rising MS4s was implemented by the course creators and faculty mentor. Ultimately, a total of 61 students participated in the course, including the 12 organizing students. Participating students received two credits—a typical distribution for medical student electives—for completion of the two-week course. The 12 students responsible for the creation of this course received an additional two elective credits for a total of four credits. The residents involved in the course graciously volunteered their time amidst their busy clinical schedules for the benefit of interested students, without receiving any credit or compensation.

### Data Collection Process

Before course enrollment, participants were asked for informed consent for analysis of their pre-assessment, post-assessment, pre- and post-lecture assignments, and other elements of course participation included in this study. This study was submitted for approval and deemed exempt by the institutional review board.

### Logistics

The course was held from April 7–May 5, 2020, and consisted of eight, 60–90 minute twice-weekly lectures. Pre-learning assignments were sent out via email at least two days prior to each class. The lectures were conducted via an online video-conference platform with sufficient bandwidth for all class participants. Post-lecture assignments were sent out immediately after the completion of the lecture and due the morning of the subsequent lecture, when answers were reviewed. Participants’ grades in the course reflected participation in lecture and completion of assignments. Assignments were not graded for accuracy.

### Pre and Post Assessments

Prior to the first lecture, enrollees completed an anonymous pre-assessment survey. Information was collected regarding their class year, previously completed clinical experiences both within and outside of EM, and self-perceived comfort with each lecture’s learning objectives. The topic-specific questions were formulated by the respective team leaders. These objectives were assessed before and after the course using a Likert scale of 1–5, ranging from “very uncomfortable” to “very comfortable.” Following the completion of the course, enrollees were sent a post-assessment survey using the same variables as the pre-assessment. We evaluated differences between mean pre-assessment and post-assessment scores using a two-tailed t-test. Significance was set as a *P*-value of less than 0.05. Additionally, they were asked to provide feedback on the course as a whole, which included the following: resident participation; student engagement; lecture style and value; and pre- and post-lecture assignments.

## IMPACT/EFFECTIVENESS

### Cohort

A total of 61 rising MS4 students attended the course, 12 of whom both attended all lectures and were responsible for leading one lecture. Of the attendees, 58 students (95%) filled out the pre-assessment survey and four students (6.9%) had just returned from a gap year between their MS3 and MS4 years. Seventeen students (29.3%) had completed an EM elective prior to this course, and 42 students (68.8% of the class participants) completed the post-assessment survey.

### Responses

**Figure 1:**
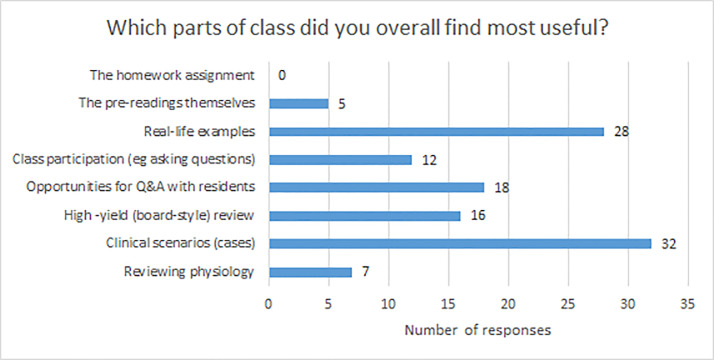
Responses to the question “Which parts of the class overall did you find most useful?” *Q&A*, question and answer.

Before the class, students were least confident in their knowledge of EM-specific clinical skills such as “conducting and interpreting the FAST exam,” “understanding indications for invasive and non-invasive ventilation,” “reading and interpreting ECGs,” and “understanding the steps of a lumbar puncture,” all of which had mean confidence scores of 2.40 or less. After the class was completed, mean confidence scores improved across all learning objectives (*P*<0.05) (Table 1). Overall, students found the clinical cases and real-life examples to be the most useful parts of the course (Figure 1). Students felt the ideal amount of resident teaching would be 40–70% of the course material, with peer instructors teaching the rest. In particular, students valued when residents provided real-life examples and shared pearls of wisdom and caveats. Based on free-text comments, students felt a good balance was achieved between peer instruction and resident instruction: peers could teach most of the “didactic” material with a focus on what is emphasized at their training level; and residents could then provide more depth when needed, caveats, and “real-life” examples to make the material come alive, and be available to answer questions.

**Figure 2:**
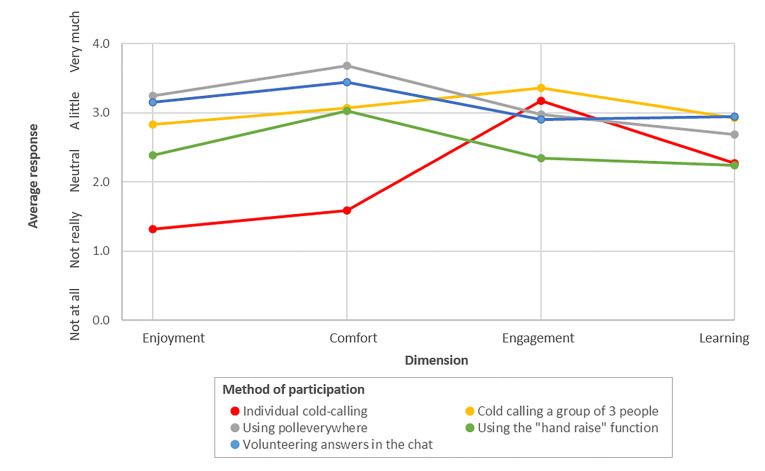
Class participation. Average response to the question “How much did [method of participation] help with your [dimension] during class?”

We assessed five different methods of class participation (Figure 2). A combination of calling on students in groups of three, online group polling software, and asking for volunteer responses in the video conference platform’s “chat” feature were found to maximize learning and engagement without sacrificing student perceived enjoyment/comfort. Cold-calling individuals, while good for engagement, was not considered as helpful for learning, and was the least enjoyable/comfortable method. Using the “hand raise” function, while comfortable, was not as engaging and did not facilitate as much learning. Based on free-text responses, individual cold-calling added stress and reduced enjoyment in the course. Students suggested that this method potentially inhibited learning because students were more focused on whether they would be called on rather than on the material itself.

Chief complaint overviews from the SAEM MS3 curriculum^18^ and podcasts were considered the most valuable types of pre-reading material. A total of 76% of respondents reported completing greater than 50% of the assigned pre-reading. Homework assignments were considered helpful, especially when they were more challenging and forced critical thinking, rather than simply testing knowledge of definitions and basics.

## DISCUSSION

The purpose of creating this novel EM curriculum was to 1) bolster the education of our peers during a period of significant disruption to our traditional clinical learning opportunities, and 2) prove the efficacy of a student-led, remote course in engaging students and increasing their knowledge base on core EM topics. Previous survey of student attitudes toward online learning during the COVID-19 pandemic demonstrated general dissatisfaction; students emphasized that currently available methods are asynchronous and that they largely prefer in-person learning due to the lack of interactive experiences available and the subsequent inability to ask questions while learning.^19^ We demonstrated that engagement can be attained on a virtual platform and that this mode of clinical education, while not ideal, was sufficient for increasing students’ perceived comfort with core clinical concepts. This methodology can be applied to situations where in-person learning is unavailable, beyond the COVID-19 pandemic.

Strengths of this project included a highly motivated group of medical students to serve simultaneously as course organizers and lecturers, support from experienced EM residents in reviewing lecture content and providing clinical pearls during presentations, and faculty to provide oversight to the course structure and lecture-specific learning objectives. The speed with which the course organizers were able to construct this novel curriculum speaks to the feasibility of recreating this model when needed.

While students generally felt satisfied with the amount and content of resident teaching during each lecture, an acknowledged challenge was deciding how to best use and standardize the residents’ input. Strategies to increase resident involvement included asking resident lecturers to monitor the “chat” of the video-conferencing platform and answer participant questions in real time, describe cases from their clinical experience relevant to the lecture topic, and explain the steps of clinical procedures included within the presentations themselves. One method to further integrate resident participation into the student-led lectures was through a “rehearsal” lecture prior to the scheduled class, such that resident-provided “clinical pearls” could be coordinated within the flow of the presentation. Limitations to implementing this across lectures included the rapid transition from course creation to presentation and the often conflicting schedules of students and residents.

Overall, zero participants found homework as the most useful aspect of the course, and very few found pre-reading most useful. Homework assignments were not graded, which has been shown to reduce perceived usefulness by Doom et al.^20^ Rather, clinical scenarios and real-life cases were rated as most useful, emphasizing the strength of the participatory, case-based aspects of the course. In future iterations of this course, further emphasis and review of pre- and post-lecture assignments may increase perceived educational value.

A key focus of this project was optimization of participation strategies to balance student comfort with engagement. While the concept of case-based learning itself is well supported in the literature, the body of evidence suggesting the optimal strategy for engaging students on a virtual platform is in its early stages of development.^16^,^17^ Morawo et al acknowledge the ease and inevitability of low engagement during virtual learning. They used polling software to demonstrate that learners were more actively engaged with the use of quizzes, both anonymously and in groups, but did not determine which modality was preferred and were limited in the modalities tested.^21^

Strategies for student engagement in this course changed throughout based on real-time feedback from participating students. Early in the course, student participation was garnered primarily in a “cold-call” fashion, in which individual students were randomly selected to answer a clinical question. While some students expressed favor with this strategy, others felt discomfort. This feedback led to use of group-based questions (ie, calling on three people simultaneously), anonymous participation (ie, using a polling software), and low stakes participation (ie, using the video conference “chat” function) for subsequent lectures. Three-person call-outs, polling, and the “chat” function are particularly effective in an online environment where participants are not always visible, and muting and unmuting can be cumbersome..

Some aspects of the course limited analysis. Attendance for each session, although near 100% based on comparison of the chat participants with the number of enrolled students in real-time, was not recorded; however, pre- and post-lecture assessments were monitored for completion and achieved a high rate of involvement. Use of anonymous and low-stakes participation limited the ability to objectively measure how many students engaged in each method; rather, analysis was based on feedback in the post-course assessment.

## CONCLUSION

Overall, the EM curriculum presented here provided valuable education to students impacted by curriculum disruptions due to the COVID-19 pandemic. With further refinement, we hope that this model for course dissemination can be adapted in other institutions to further students’ education.

## Figures and Tables

**Table 1:** Pre-course and post-course assessment of comfort with learning objectives in an online emergency medicine course led by near-peers.

Lecture	Objective	Pre-course mean	Post-course mean	Relative difference	P-value
Imaging	Reading a chest radiograph	3.03	3.73	23.0%	<0.001
	Interpreting a CT	2.57	3.51	36.6%	<0.001
	Developing a differential and using imaging to establish a diagnosis	2.98	3.83	28.3%	<0.001
Chest Pain and EKG	Using a framework to approach chest pain in the ED Setting	3.25	4.02	24.0%	<0.001
	Reading and interpreting ECGs	2.34	3.02	29.0%	<0.05
Stroke and Lumbar Puncture	Recognizing signs and symptoms of a stroke	3.72	4.29	15.3%	<0.001
	Describing initial management of an acute stroke	3.34	4.07	21.8%	<0.001
	Understanding the steps of a lumbar puncture	2.40	3.59	49.6%	<0.001
Abdominal Pain	Recognizing when emergent resuscitation is required	2.78	4.05	45.9%	<0.001
	Creating a differential diagnosis for abdominal pain	3.76	4.39	16.8%	<0.001
	Understanding the imaging work-up for abdominal pain	3.24	4.10	26.4%	<0.001
Altered Mental Status and Toxicology	Creating a differential diagnosis for altered mental status in the ED	3.19	4.27	33.8%	<0.001
	Differentiating between different presentations of common toxidromes	2.40	3.68	53.7%	<0.001
	Understanding the components of a basic toxicology screen	2.47	3.68	49.4%	<0.001
Shortness of Breath and Ventilators	Assessing shortness of breath in the ED	3.23	4.02	24.7%	<0.001
	Understanding the indications for invasive and non-invasive ventilation	2.09	3.39	62.5%	<0.001
Shock and Sepsis	Understanding how to classify shock based upon a bedside examination	2.38	3.83	60.9%	<0.001
	Identifying several causes of shock	3.14	4.22	34.5%	<0.001
Trauma and FAST Exams	Managing a trauma patient who initially arrives in the ED	2.24	3.61	61.1%	<0.001
	Conducting and interpreting the FAST exam	1.93	3.29	70.5%	<0.001

*CT*, computed tomography; *ECG*, electrocardiogram; *ED*, emergency department; *FAST*, focused assessment with sonography for trauma.
